# Protein Kinase A and 5′ AMP-Activated Protein Kinase Signaling Pathways Exert Opposite Effects on Induction of Autophagy in Luteal Cells

**DOI:** 10.3389/fcell.2021.723563

**Published:** 2021-11-08

**Authors:** Emilia Przygrodzka, Corrine F. Monaco, Michele R. Plewes, Guojuan Li, Jennifer R. Wood, Andrea S. Cupp, John S. Davis

**Affiliations:** ^1^ Department of Obstetrics and Gynecology, Olson Center for Women’s Health, University of Nebraska Medical Center, Omaha, NE, United States; ^2^ Department of Cellular and Integrative Physiology, University of Nebraska Medical Center, Omaha, NE, United States; ^3^ Veterans Affairs Nebraska Western Iowa Health Care System, Omaha, NE, United States; ^4^ Department of Animal Science, University of Nebraska-Lincoln, Lincoln, NE, United States

**Keywords:** corpus luteum, PGF2α, luteinizing hormone, AMPK, PKA, MTOR, LC3B, Ulk1

## Abstract

In the absence of pregnancy the ovarian corpus luteum undergoes regression, a process characterized by decreased production of progesterone and structural luteolysis involving apoptosis. Autophagy has been observed in the corpus luteum during luteal regression. Autophagy is a self-degradative process important for balancing sources of cellular energy at critical times in development and in response to nutrient stress, but it can also lead to apoptosis. Mechanistic target of rapamycin (MTOR) and 5′ AMP-activated protein kinase (AMPK), key players in autophagy, are known to inhibit or activate autophagy, respectively. Here, we analyzed the signaling pathways regulating the initiation of autophagy in bovine luteal cells. *In vivo* studies showed increased activating phosphorylation of AMPKα (Thr172) and elevated content of LC3B, a known marker of autophagy, in luteal tissue during PGF2α-induced luteolysis. *In vitro*, AMPK activators 1) stimulated phosphorylation of regulatory associated protein of MTOR (RPTOR) leading to decreased activity of MTOR, 2) increased phosphorylation of Unc-51-Like Kinase 1 (ULK1) and Beclin 1 (BECN1), at sites specific for AMPK and required for autophagy initiation, 3) increased levels of LC3B, and 4) enhanced colocalization of autophagosomes with lysosomes indicating elevated autophagy. In contrast, LH/PKA signaling in luteal cells 1) reduced activation of AMPKα and phosphorylation of RPTOR, 2) elevated MTOR activity, 3) stimulated phosphorylation of ULK1 at site required for ULK1 inactivation, and 4) inhibited autophagosome formation as reflected by reduced content of LC3B-II. Pretreatment with AICAR, a pharmacological activator of AMPK, inhibited LH-mediated effects on RPTOR, ULK1 and BECN1. Our results indicate that luteotrophic signaling via LH/PKA/MTOR inhibits, while luteolytic signaling via PGF2α/Ca^2+^/AMPK activates key signaling pathways involved in luteal cell autophagy.

## Introduction

The corpus luteum is a transient endocrine gland crucial for the establishment and maintenance of pregnancy. Luteinizing hormone (LH), a gonadotropin produced by the pituitary gland, regulates the formation, development, and maintenance of the corpus luteum (Davis and Rueda 2002; Berisha and Schams 2005). A main function of the corpus luteum is to produce progesterone, a steroid hormone that regulates embryo development, reduces uterine contractions, suppresses maternal immune response, prepares endometrium for implantation, prevents preeclampsia, and regulates the estrous/menstrual cycle (Davis and Rueda 2002; Park et al., 2020; Pereira et al., 2021). In the absence of an embryo, the corpus luteum undergoes regression, a process characterized by decreased production of progesterone and structural luteolysis (Juengel et al., 1993; Rueda et al., 1997; McCracken et al., 1999), ultimately resulting in the formation of a fibrotic scar called the corpus albicans. Luteolysis is triggered by a lack of gonadotropin support and/or prostaglandin F2α (PGF2α), produced within the gland and/or by the endometrium in domestic farm animals (McCracken et al., 1972; Bennegård et al., 1991; Pereira et al., 2021). If fertilization occurs, the presence of live embryos in the uterus extends the lifespan of the corpus luteum due to the luteotrophic and anti-luteolytic actions of conceptus signals (Davis and Rueda 2002).

The luteolytic action of PGF2α is mediated by binding to its G-protein coupled receptor and activating Gαq/11, which ultimately evokes multiple intracellular signals such as activation of phospholipases C and A2, elevation of intracellular calcium ions, and activation of calmodulin, protein kinase C (PKC) and mitogen-activated protein kinase (MAPK) signaling cascades (Davis et al., 1987; McCann and Flint 1993; Chen et al., 2001; Arvisais et al., 2010; Kurusu et al., 2012). These pathways trigger the activation of early response genes leading to molecular changes in the luteal tissue, consequently initiating functional and structural luteolysis (Chen et al., 2001; Talbott et al., 2017; Pate 2020). Recent studies showed that PGF2α enhances autophagy in rat, goat, and bovine luteal cells during regression (Choi et al., 2011; Aboelenain et al., 2015; Grzesiak et al., 2018; Wen et al., 2020). Moreover, the expression of genes associated with autophagy (MAP1LC3A, MAP1LC3B, ATG3, and ATG7) and the protein content of microtubule associated protein 1 light chain 3 beta (MAP1LC3B commonly referred to as LC3B), a known marker of the formation of autophagosomal vacuoles, increased in the bovine corpus luteum during the late luteal phase in comparison to the mid-luteal phase (Aboelenain et al., 2015). Similar changes were observed in the corpus luteum of mice during heat stress (Ullah et al., 2019).

Autophagy is the so called “self-eating” process during which cells digest damaged or old organelles in order to recycle intracellular content providing building blocks required to maintain basic cellular functions (Parzych and Klionsky 2014). Due to the different types of cargo digested, autophagy is divided into microautophagy, chaperone-mediated autophagy, and macroautophagy. The best characterized type of autophagy is macroautophagy, which is initiated by isolation of the targeted cytoplasmic constituents from the rest of the cell within a double-membraned vesicle known as an autophagosome. Mature autophagosomes are transported to the perinuclear region where they fuse with lysosomes leading to degradation of the autophagosome cargo (Parzych and Klionsky 2014). Although autophagy is often considered as a pro-survival process, there is growing body of evidence that autophagy can lead to apoptosis (Song et al., 2018; Denton and Kumar 2019).

The process of autophagy is oppositely regulated by two main intracellular protein kinases i.e., 5’ AMP-activated protein kinase (AMPK) and mechanistic target of rapamycin kinase (MTOR) (Yang and Klionsky 2009; He et al., 2018; Wang and Zhang 2019). AMPK activates autophagy at different posttranscriptional levels including 1) inactivation of MTOR via phosphorylation of regulatory-associated protein of MTOR complex 1 (RPTOR) and 2) direct activation of proteins related to autophagosome formation such as Unc-51-Like Kinase 1, ULK1, a mammalian orthologue of Atg1 or Beclin-1 (BECN1) (Gwinn et al., 2008; Kim et al., 2011; Kim et al., 2013). In addition, AMPK can also regulate the activities of transcription factor EB (TFEB) and forkhead box O3 (FOXO3), transcription factors governing expression of autophagic genes (Yang and Klionsky 2009). In contrast, MTOR inhibits autophagy by phosphorylation of ULK1 at Ser757, which inhibits the kinase activity of the ULK1 complex via disruption of the interaction between ULK1 and AMPK (Egan et al., 2011; Kim et al., 2011). Thus, AMPK and MTOR signaling exert opposite regulatory effects on the induction of autophagy.

In the ovary, autophagy has been associated with follicle development and atresia as well as luteal formation and regression (Escobar et al., 2008; Choi et al., 2011; Aboelenain et al., 2015; Grzesiak et al., 2018; Meng et al., 2018; Zhang et al., 2018; Tang et al., 2019; Tang et al., 2020; Wen et al., 2020). However, the signaling pathways responsible for regulation of autophagy in luteal cells have received little attention. Previous studies established that LH, the main hormone regulating formation and maintenance of the corpus luteum, decreases activity of AMPK and increases the activity of MTOR via protein kinase A (PKA) signaling in bovine luteal cells (Hou, Arvisais and Davis 2010; Tang et al., 2020; Przygrodzka et al., 2021). In contrast, the luteolytic hormone PGF2α stimulates AMPK activity in response to elevations in intracellular calcium at the time of luteolysis (Bowdridge et al., 2015). Thus, we hypothesize that PKA/MTOR signaling and AMPK signaling exert opposite effects on proteins involved in autophagy induction in luteal cells. The aims of the present study were to determine: 1) the acute effects of AMPK on phosphorylation of key autophagy initiating proteins (RPTOR, ULK1, BECN1); 2) the role of PKA signaling on the phosphorylation of MTOR, AMPK, RPTOR and ULK1, as well as the content of LC3B-II, a marker of autophagy; and 3) the effects of AMPK on LH-stimulated phosphorylation of RPTOR and BECN1 in luteal cells.

## Materials and Methods

### Reagents

Antibodies used for this study are listed in Table 1. M199 and fetal bovine serum were from Cambrex (Walkersville, MD, United States). Type II collagenase was obtained from Atlantic Biologicals (Lawrenceville, GA, United States). Compound C (AMPK inhibitor) was obtained from Millipore Corporation (Burlington, MA, United States). PGF2α was purchased from Cayman Chemical Company (Ann Arbor, MI, United States). Forskolin (adenylyl cyclase activator), and Rapamycin (MTOR inhibitor) were purchased from EMD Chemicals, Inc. (Gibbstown, NJ, United States). H89 (PKA inhibitor) and chloroquine (lysosome inhibitor), were obtained from Tocris Bioscience (Minneapolis, MN, United States). Bovine LH was purchased from Tucker Endocrine Research Institute (Atlanta, GA). 5-Aminoimidazole-4-carboxamide-1-β-4-ribofuranoside (AICAR, AMPK activator) was from Toronto Research Chemicals Inc. (Toronto, Ontario, Canada). Protease and phosphatase inhibitor cocktails, CYTO-ID autophagy kit 2.0 was purchased from Enzo Life Sciences Inc. (Farmindale, NY, United States) (Guo et al., 2015). Organelle specific dyes (Mitotracker Deep Red, Lysotracker, LipiBlue) and Pierce ECL Western Blotting Substrate were purchased from Thermo Fisher Scientific (Waltham, MA, United States). The protein assay kit was purchased from Bio-Rad Laboratory (Richmond, CA, United States). Nitrocellulose membranes for western blotting were from Millipore (Bedford, MA, United States). The adenoviruses encoding dominant-negative HA-tagged AMPKα1 (Ad.dn.AMPK) with a D159A mutation in the ATP-binding domain were purchased from Eton Bioscience (San Diego, CA, United States) and used as previously described (Przygrodzka et al., 2021). The AMPKα1 mutant cannot bind to ATP and competes with wild-type AMPK for binding with the β and γ subunits. The control adenoviruses with cytomegalo virus promoter (Ad-Empty) were used as previously described (Taurin et al., 2008).

**TABLE 1:** Antibodies Used in this Study.

Antibody name	Species specificity	Source	Dilution	Catalog number	Company
phospho-BECLIN Ser93	Mouse	Rabbit pAB	1:1,000	14717	Cell Signaling Technology (Beverly, MA, United States)
phospho-RAPTOR Ser792	Mouse	Rabbit pAB	1:1,000	2083	Cell Signaling Technology (Beverly, MA, United States)
phospho-ULK1 Ser757	Mouse	Rabbit pAB	1:1,000	6888	Cell Signaling Technology (Beverly, MA, United States)
phospho-ULK1 Ser317	Mouse	Rabbit pAB	1:1,000	37762	Cell Signaling Technology (Beverly, MA, United States)
Ulk1	Mouse	Rabbit mAB	1:1,000	8054	Cell Signaling (Baverly, MA, United States)
phospho-MTOR Ser2448	Mouse	Rabbit pAB	1:1,000	2917	Cell Signaling Technology (Beverly, MA, United States)
phospho-AMPK Thr172	Mouse	Rabbit pAB	1:1,000	2535	Cell Signaling Technology (Beverly, MA, United States)
phospho-AMPK Ser485	Mouse	Rabbit pAB	1:1,000	4185	Cell Signaling Technology (Beverly, MA, United States)
BECN1	Mouse	Rabbit pAB	1:1,000	3738	Cell Signaling (Baverly, MA, United States)
LC3B	Mouse	Rabbit mAB	1:1,000	3868	Cell Signaling Technology (Beverly, MA, United States)
ACTB	Bovine	Mouse mAB	1:10,000	A5441	Sigma Chemical Co. (St. Louis, MO, United States)
HRP linked	Anti-rabbit	Goat pAB	1:10,000	111035003	Jackson Research Laboratory (West Grove, PA, United States)
HRP linked	Anti-mouse	Goat pAB	1:10,000	115035205	Jackson Research Laboratory (West Grove, PA, United States)
phospho-p70S6K Thr 389	Mouse	Rabbit mAB	1:1,000	9234	Cell Signaling (Baverly, MA, United States)
phospho-ERK1/2 Thr202/Tyr204	Mouse	Rabbit pAB	1:1,000	9101	Cell Signaling (Baverly, MA, United States)

### Animal Preparation, *in vivo* Experiments and Ovariectomies

All animal procedures were approved by the IACUC at the University of Nebraska-Lincoln. Non-lactating crossbred beef cows between 2 and 6 years in age (Red Angus, Pinsgauer, Red Poll, Herford composites with some Red Angus/Limousin) from the UNL Physiology research and teaching herds were used. To synchronize animal estrous cycles PGF2α (25mg; Lutalyse®, Zoetis Inc., Kalamazoo Michigan, MI) was injected on Day 1 with a subsequent injection of GnRH 36 h later. Ovulation occurs 24 h post-GnRH injection, and on day 13.5 cows will have fully functional mid-luteal phase corpus luteum, equivalent to day 10 in the natural cycle. Cows were fasted for 12–18 h before ovariectomy but retained ad libitum access to water. At day 13.5 cows were treated with an intra-muscular injection of saline (n = 3) or PGF2α (25mg; Lutalyse®) (n = 3). Transrectal ultrasonography was performed immediately before ovariectomy to confirm the presence of corpus luteum. Ovaries were removed via a right-flank approach paralumbar fossa laparotomy, to avoid the rumen and prevent internal hemorrhaging, as previously detailed (Summers et al., 2014; Talbott et al., 2017). Portions of luteal tissue were snap frozen using liquid nitrogen for subsequent protein analysis.

### Isolation and Culture of Bovine Luteal Cells

Cells were isolated as previously described (Hou et al., 2010; Przygrodzka et al., 2021). Ovaries of early pregnant cows (fetal crown rump length <12 cm) were obtained from a local abattoir (JBS, Omaha, NE) and transported to the laboratory in cold M199, on ice. Corpora lutea were sliced and dissociated with collagenase (1.03 U/mL). Small and large luteal cells were separated by centrifugal elutriation as previously described (Hou et al., 2010), which were then cultured overnight on tissue culture plates in basal medium [M199 containing 0.1% bovine serum albumin (BSA) and antibiotics] with 5% fetal bovine serum at 37°C in a humidified atmosphere of 5% CO_2_. Two hours prior to the addition of treatments, the medium was removed, cells were washed in warm phosphate-buffered saline (PBS) and equilibrated in serum-free basal medium.

### Western Blot Analysis

Cell lysis and Western blot analysis were performed as previously published (Hou et al., 2010). Lysates were subjected to separation on 10% SDS-PAGE (30 μg protein per lane) and transferred onto nitrocellulose membranes, which were then blocked with 5% BSA in Tris-buffered saline with 0.1% Tween-20 (TBST) at room temperature for 1 h. Then, membranes were incubated with primary antibodies (Table 1) at 4°C overnight. Membranes were washed with TBST and blocked in secondary HRP-conjugated antibodies (1: 10,000) in 2.5% non-fat milk in TBST for 1 h, followed by a second series of washes. Blots were imaged and quantified using a Biospectrum Imaging System.

### Immunostaining and Confocal Microscopy

Confocal microscopy was used to evaluate the effects of LH and AICAR on the presence and regulation of autophagosomes in bovine luteal cells. Mixed luteal cells were seeded at a density of 3 × 10^5^ cells/well on cell culture imaging dishes equipped with #1.5 glass bottom slide and maintained at 37°C in an atmosphere of 95% humidified air and 5% CO_2_. Adhered luteal cells were equilibrated in fresh culture medium enriched with 1% BSA for 2 h prior to stimulation with LH (10 ng/ml) or AICAR (1 mM) for 3 h. Organelle specific dyes, CYTO-ID® Green Detection Reagent (autophagosomes), Mitotracker Deep Red (mitochondria), Lysotracker (lysosomes), and LipiBlue (lipid droplets) were added to each well according to manufacturer’s protocol and maintained at 37°C in an atmosphere of 95% humidified air and 5% CO_2_ for 30 min. Following incubation, cells were fixed for 30 min with 200 µl 4% paraformaldehyde at 4°C and rinsed three times with PBS.

Images were collected using a Zeiss LSM800 confocal microscope with Airyscan equipped with a 20× (0.8 N.A) and ×63 oil immersion objective (1.4 N.A) and acquisition image size of 1,024 × 1,024 pixel (245.73 µm × 245.73 µm for 20×; 78.01 µm × 78.01 µm for 63×). The appropriate filters were used to excite each fluorophore and emission of light was collected between 450 and 1,000 nm. Cells were randomly selected, and z-stacked (0.3 µm) images were generated from bottom to top of each experiment. To determine the effects of LH or AICAR on mean fluorescence intensity of CYTO-ID® Green Detection Reagent, images were converted to maximum intensity projections and processed utilizing ImageJ (National Institutes of Health) analysis software. Mean fluorescence intensity was determined as previously described (Plewes et al., 2020; Talbott et al., 2020).

### Statistical Analysis

The data are presented as the means ± standard error of the mean (SEM) from multiple experiments (at least 3 independent experiments from different animals). GraphPad Prism v. 8.0 software was used for statistical analysis. Data were analyzed by either Student’s t-test, or one-way analysis of variance (ANOVA) followed by Bonferroni multiple comparison tests. Statistical significance was determined as *p < 0.05*.

## Results

### Phosphorylation of AMP-Activated Protein Kinaseα and Content of Autophagy Marker Increase in the Corpus Luteum During Luteolysis

**FIGURE 1:**
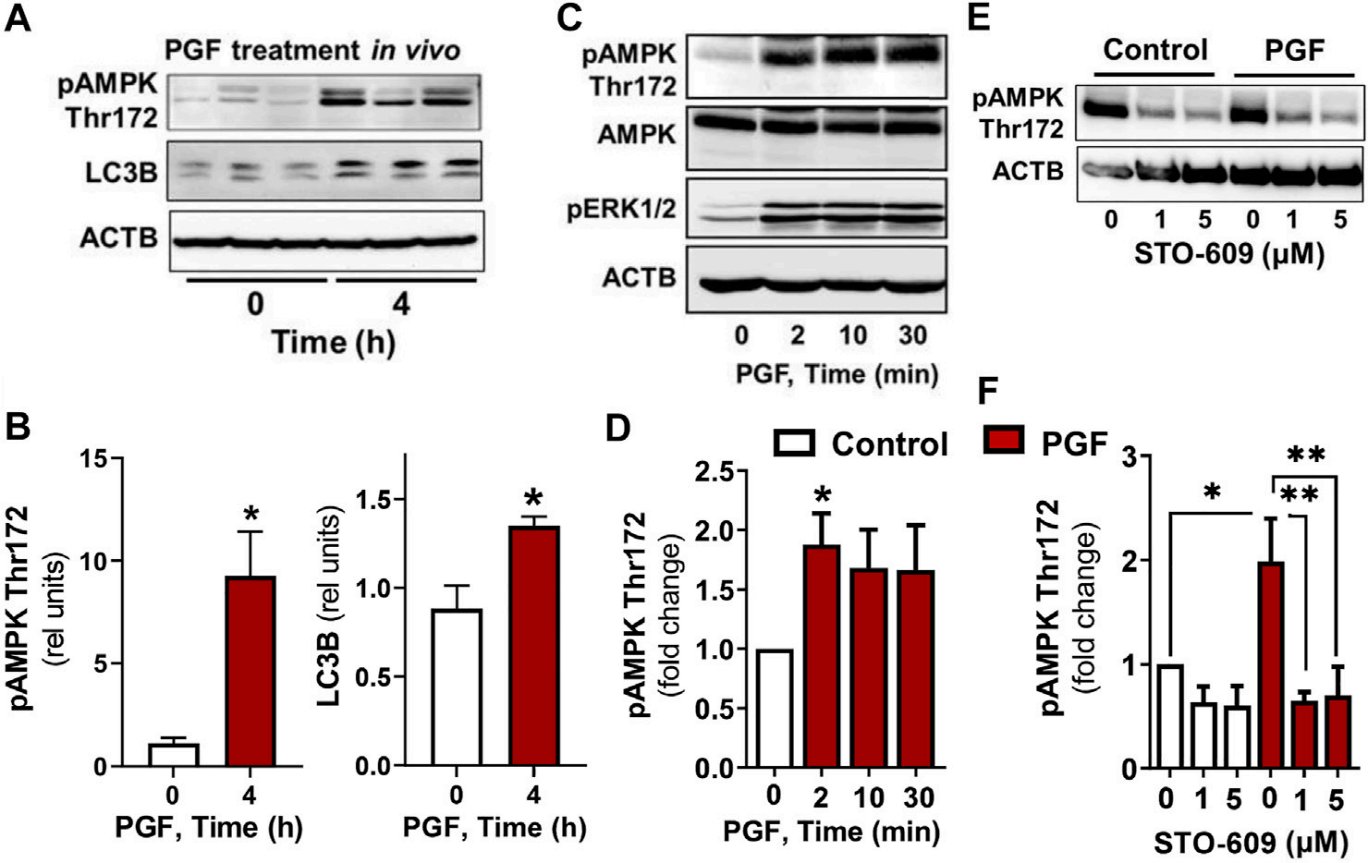
AMPK activity and markers of autophagy increase in the bovine corpus luteum during luteal regression. Representative blots **(A)** and bar graphs **(B)** showing phosphorylation of AMPKα (Thr172) and content of LC3B in luteal tissue post-PGF2α injection *in vivo*. **(C–F)** Primary cultures of bovine small luteal cells were treated with PGF2α or pretreated with inhibitor of CAMKK2 (STO-609) and then treated with PGF2α (100 nM). Representative blots **(C, E)** and bar graphs **(D, F)** showing phosphorylation of AMPKα (Thr172), ERK1/2, and total AMPKα in the luteal cells. Data are shown as a relative units or fold change and presented as means ± SEM from three-four separate experiments. Data were normalized to β-actin (ACTB). Statistical analysis was performed using student’s t-test or one-way ANOVA test with post hoc Bonferroni test. *, ** as *p* < 0.05 and *p* < 0.01.

*In vivo* experiments showed an elevated (*p < 0.05*) phosphorylation of AMPKα at Thr172, a site required for AMPK activity, in luteal tissue post-PGF2α injection. Simultaneously, we observed increased (*p < 0.05*) content of LC3B in luteal tissue of cows injected with PGF2α in comparison to untreated animals (Figures 1A,B).

### Prostaglandin F2α/Calmodulin Dependent Protein Kinase Kinase 2 Simulates Phosphorylation of AMP-Activated Protein Kinaseα

To confirm that PGF2α triggers AMPK, bovine large luteal cells were incubated in the presence of PGF2α for 2–30 min. We observed a rapid increase (*p < 0.05*) in phosphorylation of AMPKα Thr172 in cells incubated with PGF2α (Figures 1C,D). Enhanced phosphorylation of ERK (extracellular signal-regulated protein kinase) was observed in response to PGF2α (Figure 1C), confirming effective activation of PGF2α receptors. Knowing that AMPK can be activated by calcium/calmodulin dependent protein kinase kinase 2 (CAMKK2), a kinase evoked by PGF2α in bovine luteal tissue (Davis et al., 1987; Bowdridge et al., 2015; Herzig and Shaw 2018), we pretreated luteal cells with inhibitor of CAMKK2, STO-609, and then treated them with PGF2α. Preincubation with STO-609 abolished the stimulatory effect of PGF2α on AMPKα phosphorylation (Figures 1E,F).

### AMP-Activated Protein Kinase Stimulates Phosphorylation of Proteins Required for Mechanistic Target of Rapamycin Kinase Inhibition and Autophagy Initiation

**FIGURE 2:**
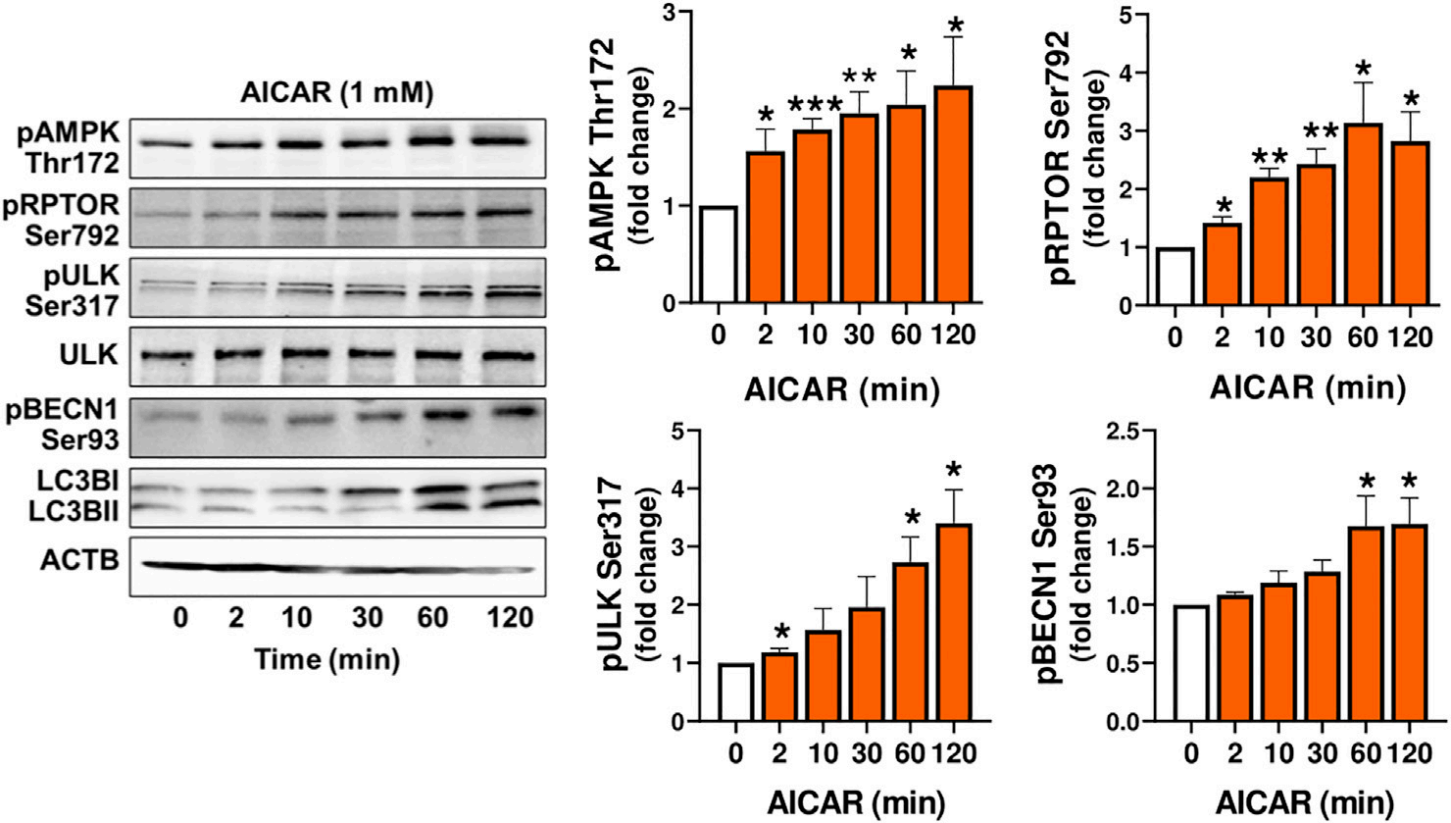
The AMPK activator AICAR triggers phosphorylation of proteins involved in MTOR inhibition and autophagy initiation. Primary cultures of bovine small luteal cells were treated with the AMPK activator AICAR (1 mM), for 2–120 min. Representative immunoblots and bar graphs showing phosphorylation of AMPKα (Thr172), the AMPK substrates RPTOR (Ser792), ULK1 (Ser317) and BECN1 (Ser93), and total ULK1 and LC3B. Data are shown as a fold change and presented as means ± SEM from three-four separate experiments. Data were normalized to β-actin (ACTB). Statistical analysis was performed using one-way ANOVA test with post hoc Bonferroni test. *, **, *** as *p* < 0.05, *p* < 0.01 and *p* < 0.001.

Experiments were conducted to determine whether AMPK stimulates the phosphorylation of proteins involved in the induction of autophagy (RPTOR, ULK1 and BECN1) in luteal cells. Treatment of luteal cells with AICAR (1 mM) induced (*p < 0.001*) time-dependent phosphorylation of the activation loop of AMPKα (Thr172) in comparison with control cells confirming the effective action of AMPK activity in the luteal cells. Simultaneously, AICAR rapidly increased (*p < 0.01*) phosphorylation of RPTOR at Ser792, a site that inhibits MTOR activation (Gwinn et al., 2008). The AICAR-induced RPTOR phosphorylation persisted for over 120 min in small luteal cells. Treatment with AICAR also stimulated phosphorylation of ULK1 (Ser317) and BECN1 (Ser93) in luteal cells (Figure 2), both of which contribute to initiation of autophagy (Kim et al., 2011; Kim et al., 2013). We also determined elevated content of LC3B in cells treated with AICAR (Figure 2).

**FIGURE 3:**
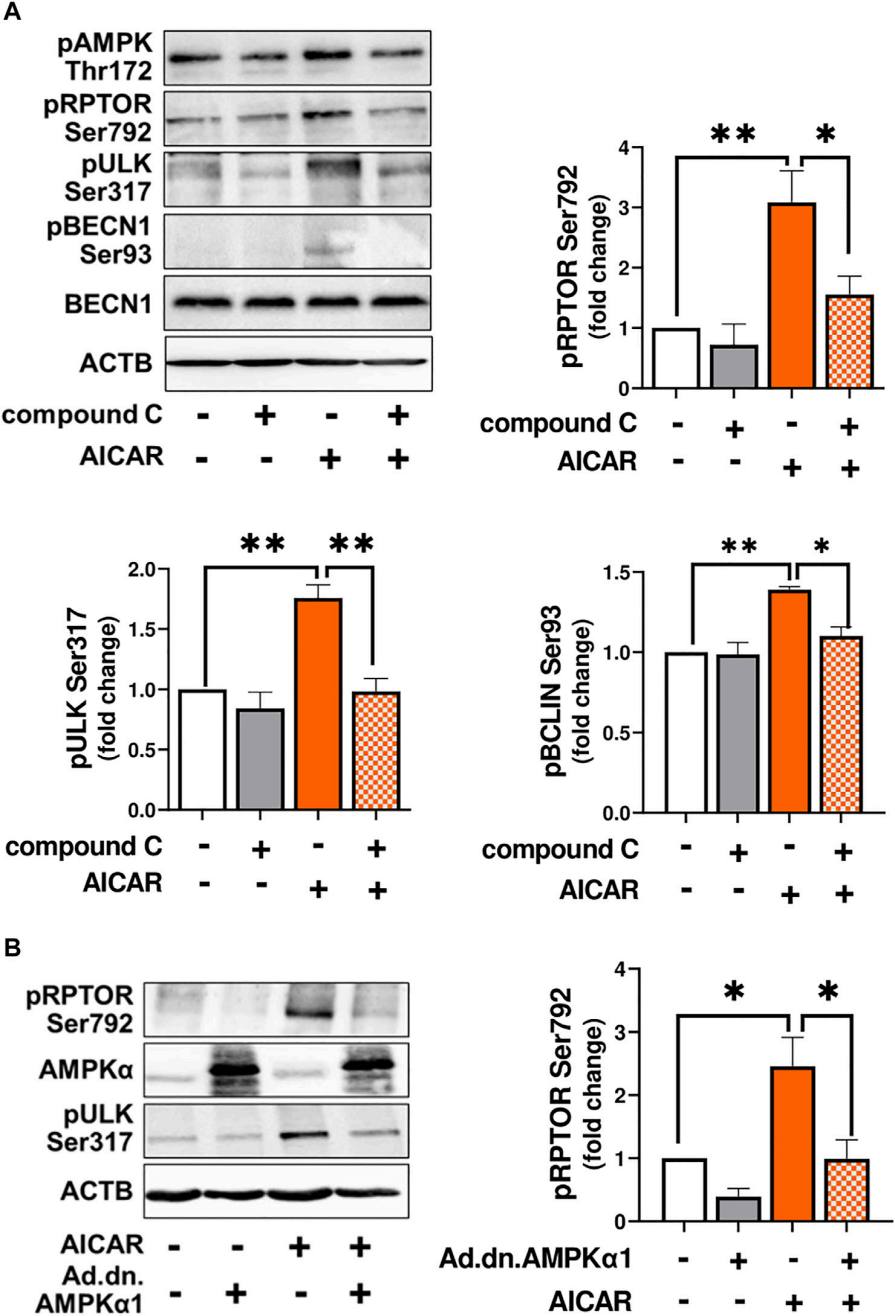
AMPK triggers post-translational modification of proteins involved in autophagy initiation. **(A)** Small luteal cells were pretreated with compound C (50 μM) for 1 h and then treated with AICAR (1 mM) for 15 min. Bar graphs and representative blots showing levels of phospho-AMPKα (Thr172), RPTOR (Ser792), ULK1 (Ser317), BECN1 (Ser93) and total BECN1. **(B)** Small luteal cells were infected with control adenovirus (Ad.CMV) or adenovirus expressing dominant negative AMPKα1 (Ad.dn.AMPKα1) for 48 h and then treated with AICAR (1 mM) for 15 min. Bar graphs and representative blots showing levels of phospho-RPTOR (Ser792), ULK1 (Ser317) and total AMPKα. Data are shown as a fold change and presented as means ± SEM from three-four separate experiments. Data were normalized to β-actin (ACTB). Statistical analysis was performed using one-way ANOVA test with post hoc Bonferroni test. Significant differences between treatments are indicated with asterisks * and ** reflecting *p* < 0.05 and *p* < 0.01.

In order to demonstrate that the response to AICAR required the activation of AMPK, luteal cells were pretreated with compound C, an inhibitor of AMPK, or transfected with dominant negative AMPK (Ad.dn.AMPKα1) and then treated with AICAR. We confirmed enhanced phosphorylation of AMPK, RPTOR, ULK1 and BECN1 in cells treated with AICAR (Figure 3A). Pretreatment with compound C abolished AICAR-mediated effects on phosphorylation of AMPK, RPTOR, ULK1, and BECN1. In addition, transfection of cells with Ad. dn.AMPKα1, which blocks catalytic activity of AMPK, prevented phosphorylation of RPTOR at Ser792 in cells treated with AICAR (Figure 3B). Thus, the effects of AICAR on phosphorylation of autophagy related proteins RPTOR, ULK1 and BECN1 are mediated via activation of AMPK.

### Luteinizing Hormone Inhibits AMP-Activated Protein Kinase Activity but Stimulates Activity of Mechanistic Target of Rapamycin Kinase

**FIGURE 4:**
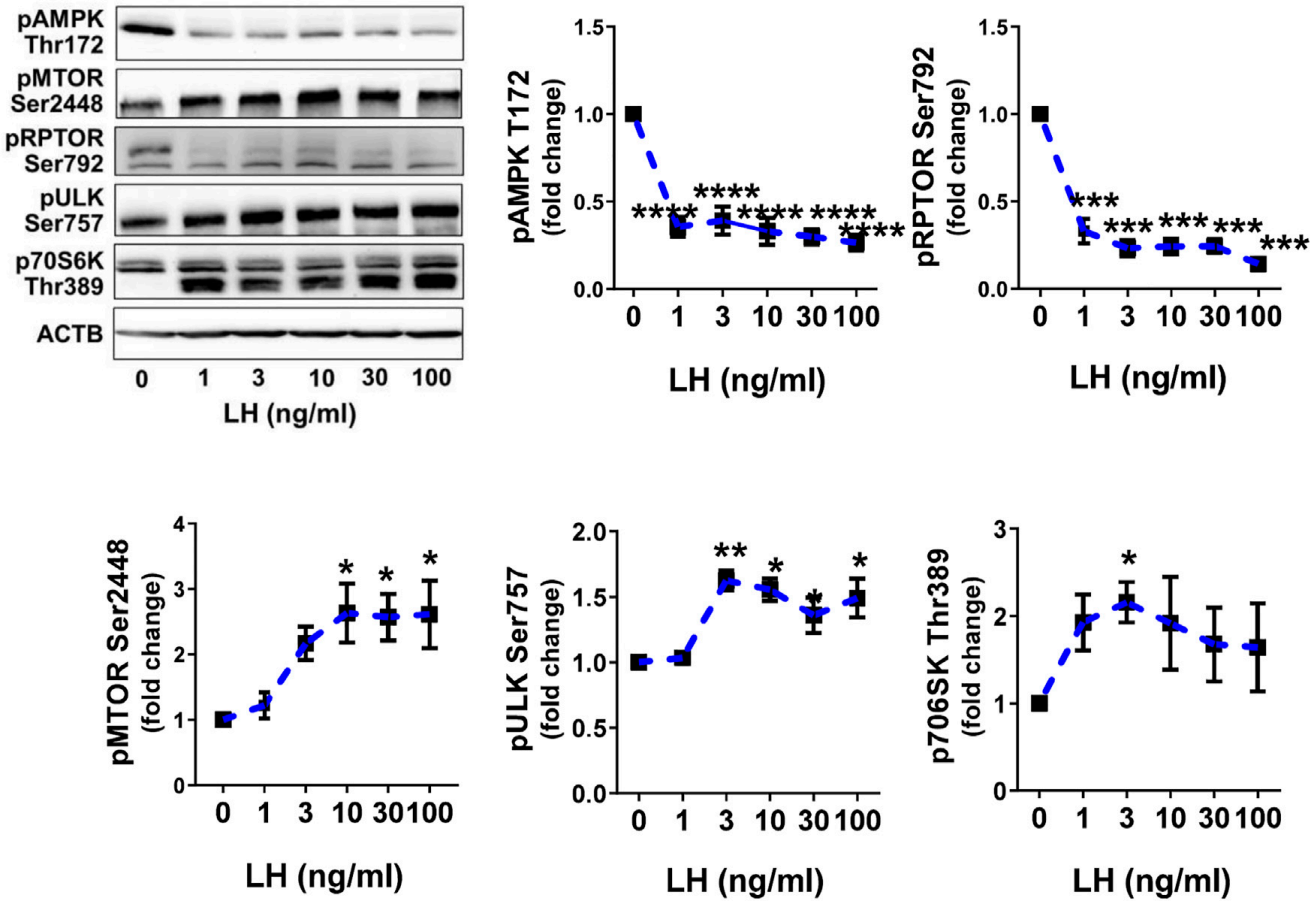
LH inhibits AMPK activity and stimulates activity of MTOR. Primary cultures of small luteal cells were treated with increasing concentrations of LH (0–100 ng/ml) for 30 min. Line graphs and representative blots showing phosphorylation of AMPKα (Thr172), RPTOR (Ser792), MTOR (Ser2448), ULK1 (Ser757) and p70S6K (Thr389). Data are shown as the average fold changes ±SEM from 3 to 5 separate experiments. Data were normalized to β-actin (ACTB). Statistical analysis was performed using one-way ANOVA with post hoc Bonferroni tests. Significant differences between LH concentrations and control are indicated with asterisks *, **, ***, **** reflecting *p* < 0.05, *p* < 0.01, *p* < 0.001, *p* < 0.0001.

Previously, we reported that LH can inhibit the phosphorylation of AMPKα and activate MTOR in bovine luteal cells (Hou et al., 2010; Przygrodzka et al., 2021). We performed studies to determine the effects of LH on phosphorylation of AMPKα, MTOR and their targets. Treatment with increasing concentrations of LH provokes concentration dependent effects of phosphorylation of AMPKα, RPTOR, MTOR, ULK1 and p70S6K (Figure 4). The lowest concentrations of LH decreased (*p < 0.001*) activating phosphorylation of AMPKα (Thr172) by 70%. Simultaneously, the LH reduced (*p < 0.001*) phosphorylation of the AMPK substrate RPTOR (Ser792) by 67%. Increasing concentrations of LH provoked concentration-dependent elevations (*p < 0.01*) in the phosphorylation of MTOR (Ser2448) and two MTOR substrates, ULK1 (Ser757) and p70S6K (Thr389) in small luteal cells (Figure 4) reflecting elevated activity of MTOR.

### Luteinizing Hormone/Protein Kinase A/Mechanistic Target of Rapamycin Kinase Signaling Affects Phosphorylation of Proteins Involved in Autophagy Initiation

**FIGURE 5:**
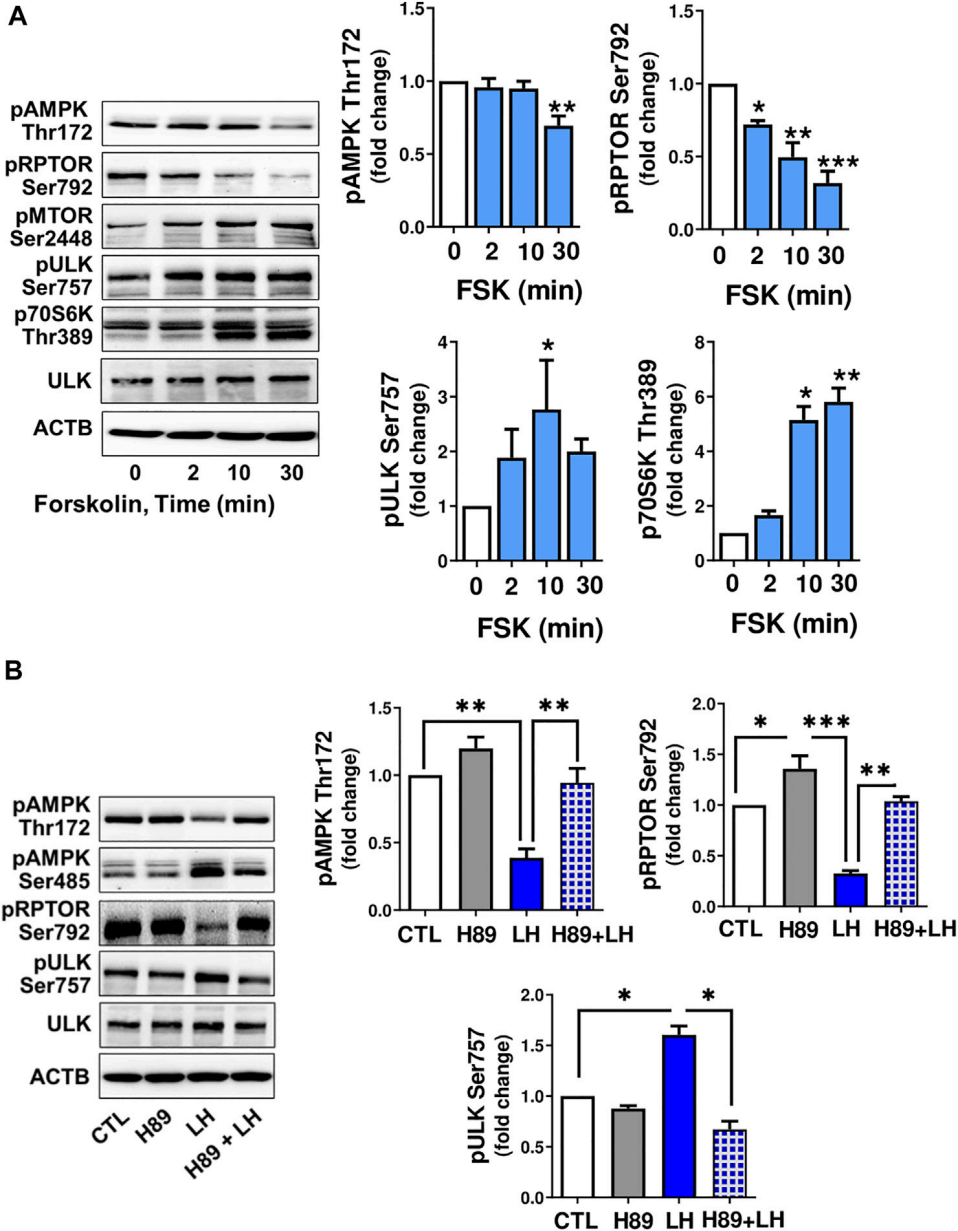
LH/PKA signaling affects phosphorylation of proteins involved in MTOR signaling or autophagy initiation. **(A)** Small luteal cells were treated with forskolin (FSK; 10 µM) for 2–30 min. Western blot analysis was performed to evaluate phosphorylation of AMPKα (Thr172 and Ser485), RPTOR (Ser792), ULK1 (Ser757) and p70S6K (Thr389). **(B)** Small luteal cells were pretreated with PKA inhibitor H89 (30 μM) for 45 min and then treated with LH (10 ng/ml) for 10 min. Representative blots present phosphorylation of AMPKα (Thr172 and Ser485), RPTOR (Ser792), ULK1 (Ser757), p70S6K (Thr389) and total ULK1. Bar graphs show average fold changes ±SEM from 3 to 5 separate experiments. Data were normalized to β-actin. Statistical analysis was performed using one-way ANOVA test for repeated measurements and Bonferroni post hoc test. Significant differences are indicated with asterisks *, ** and *** representing *p* < 0.05, *p* < 0.01 and *p* < 0.001.

Cyclic AMP/PKA signaling is the main pathway triggered by LH in luteal cells, thus small luteal cells were treated with forskolin (FSK; 10 µM), an activator of adenylyl cyclase. Similar to LH, treatment with FSK reduced (*p < 0.01*) phosphorylation of AMPKα (Thr172) and RPTOR (Ser792), a site phosphorylated by AMPK. Forskolin elevated (*p < 0.05*) phosphorylation of AMPK (Ser485), a site specific for PKA and associated with reduced AMPK activity. FSK also enhanced phosphorylation of MTOR (Ser2448) and its substrates ULK1 (Ser757) and p70S6K (Thr389) (Figure 5A).

To determine whether PKA was involved in the response to LH, small luteal cells were pretreated with a PKA inhibitor, H89 (30 μM), and then treated with LH for 10 min. Treatment with LH decreased (*p < 0.01*) phosphorylation of AMPKα at Thr172 and RPTOR at Ser792, but elevated (*p < 0.01*) phosphorylation of ULK1 at Ser757. In contrast, treatment with the PKA inhibitor H89 abrogated effects of LH on phosphorylation of AMPKα, RPTOR and ULK1 (Figure 5B) confirming LH/PKA mediated effects on AMPKα and MTOR as well their substrates in the luteal cells.

**FIGURE 6:**
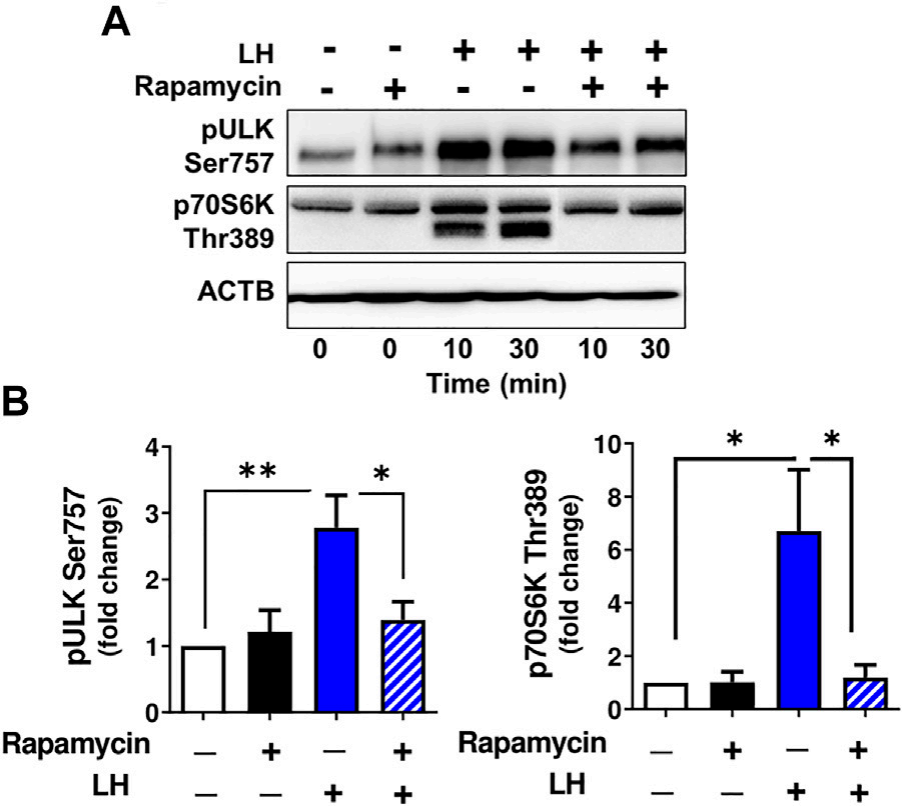
MTOR signaling triggers ULK1 phosphorylation. Small luteal cells were pretreated with MTOR inhibitor rapamycin (50 nM) for 1 h and then treated with LH (10 ng/ml) for 10 and 30 min. **(A)** Representative immunoblots showing phosphorylation of ULK1 (Ser757) and p70S6K (Thr389). **(B)** Bar graphs showing phosphorylation of ULK1 (Ser757) and p70S6K (Thr389). Data were normalized to β-actin. Statistical analysis was performed using one-way ANOVA test for repeated measurements and Bonferroni post hoc test. Significant differences are indicated with asterisks *, ** representing *p* < 0.05, *p* < 0.01.

The activity of ULK1, a protein associated with autophagosome formation (Hara et al., 2008), can be inhibited by MTOR via phosphorylation at Ser757 (Kim et al., 2011). To confirm that LH/MTOR signaling triggers phosphorylation of ULK1 at Ser757 in the luteal cells, luteal cells were pretreated with the MTOR inhibitor rapamycin (50 nM), and then treated with LH. Incubation in the presence of LH increased (*p < 0.05*) phosphorylation of ULK1 (Ser757), but pretreatment with rapamycin prevented LH-mediated effects on phosphorylation of ULK1 (Figures 6A,B) confirming that LH stimulates ULK1 phosphorylation via MTOR signaling. Pretreatment with rapamycin also abolished phosphorylation of p70S6K (Thr389) confirming effective inhibition of MTOR activity.

### Luteinizing Hormone Decreases Content of LC3B-II Indicating Inhibition of Autophagosome Formation

**FIGURE 7:**
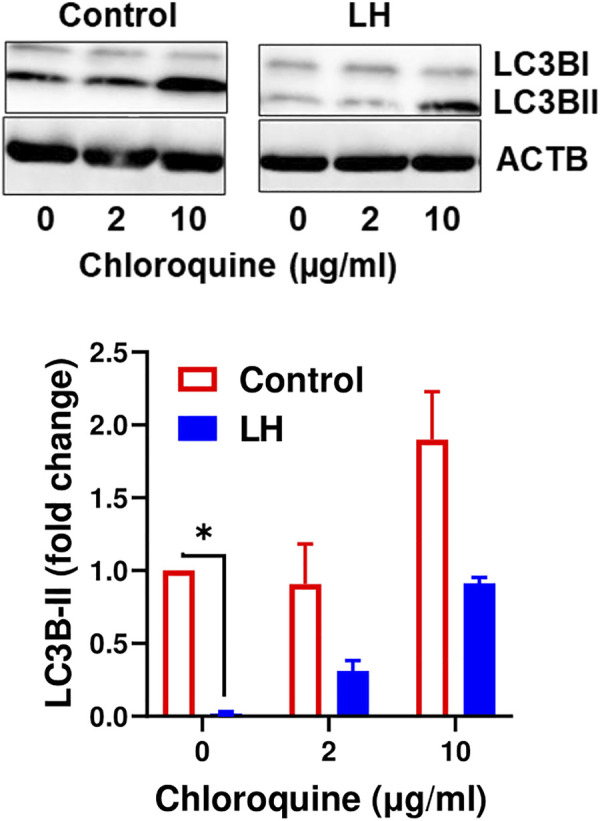
LH inhibits formation of autophagosomes. Cultures of small luteal cells were pretreated with chloroquine (2 and 10 μg/ml) for 1 h and then treated with LH (10 ng/ml) for 4 h. Representative blots and bar graphs show content of LC3B. Data are shown as average fold changes ±SEM from three separate experiments. Statistical analysis was performed using one-way with post hoc Bonferroni test. Significant differences are indicated with asterisks * as *p* < 0.05.

It is well established that LC3B serves as a specific marker for autophagosomes because the protein level of LC3B-II positively correlates to autophagosome production (Tanida et al., 2008). Because the current findings indicate that LH has an inhibitory effect on proteins involved in autophagy initiation, we tested the effect of LH on LC3B-II. Luteal cells were incubated in the presence and absence of chloroquine, an inhibitor of lysosome fusion with autophagosomes. Chloroquine elevated content of LC3B-II, which confirmed inhibition of lysosomal activity in luteal cells. In contrast, LH decreased (*p < 0.05*) content of LC3B-II in comparison to untreated cells and cells pretreated with chloroquine (Figure 7), indicating that LH inhibits autophagosome formation.

### Activation of AMP-Activated Protein Kinase Increases Colocalization of Autophagosomes With Lysosomes

**FIGURE 8:**
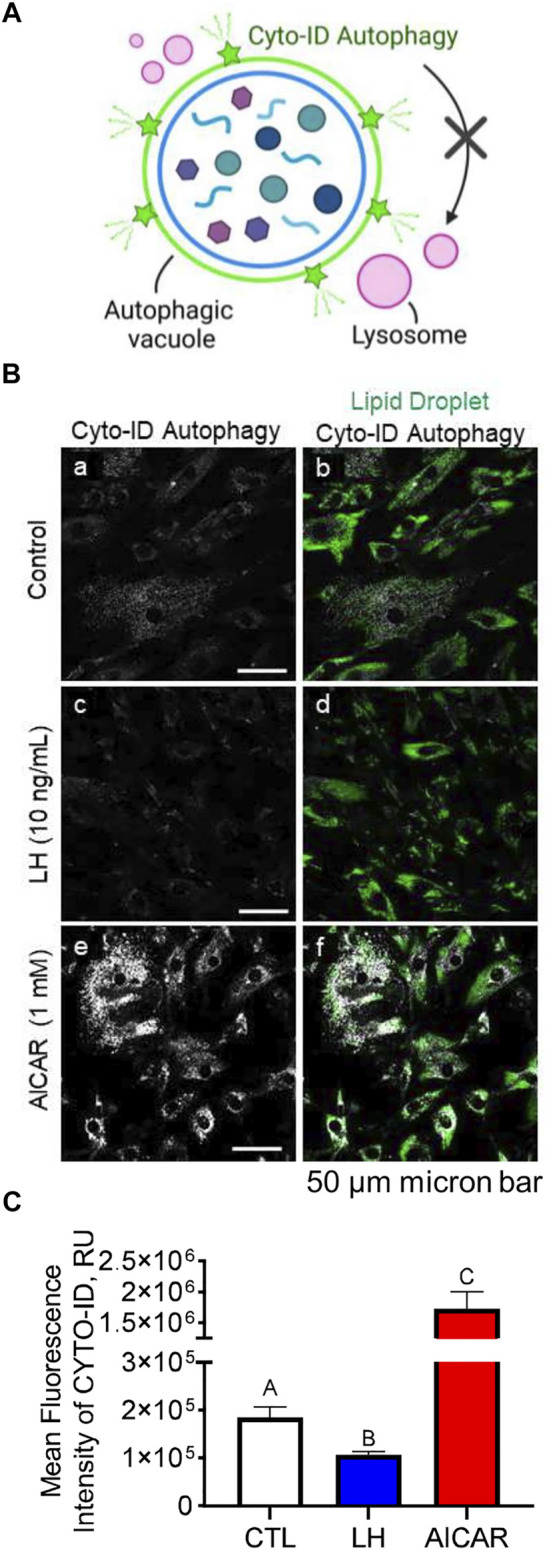
Effects Luteinizing hormone (LH) and AICAR on autophagy *in vitro*. Luteal cells were stimulated with LH (10 ng/ml) or AICAR (1 mM) for 3 h and subject to confocal microscopy. **(A)** Illustration of Cyto-ID green detection reagent mechanism of action. Cyto-ID is a cationic amphiphilic tracer dye that rapidly partitions into intracellular autophagic vacuoles. The dye exhibits unique functional moieties that prevent accumulation within lysosomes, while labeling vacuoles associated with the autophagy pathway. **(B)** Representative micrographs showing the effects of LH or AICAR on autophagy in luteal cells. From left to right; Cyto-ID autophagy (white; panels a, c and e) and merge of Cyto-ID with Lipid droplets (Lipi-Blue; green; panels b, d and f) obtained from cells treated (from top to bottom) with vehicle control, LH (10 ng/ml) or AICAR (1 mM). **(C)** Quantitative analyses of the mean fluorescence intensity normalized to cell area (MFI; relative units; RU) of Cyto-ID green detection reagent. Open bars represent control cells; closed blue bar represents cells treated with LH; closed red bar represents cells treated with AICAR. ^ABC^ Differ significantly between treatment (*p* < 0.05). Micron bar represents 50 µm.

We conducted experiments to determine the effects of LH/PKA or AMPK signaling on the colocalization of mitochondria or lysosomes with autophagosomes in luteal cells. Luteal cells were treated with AICAR or LH for 3 h and stained to observe colocalization of lipid droplets or lysosomes with autophagosomes (Figure 8A). Cyto-ID, a recently developed cationic amphilic tracer dye with minimal staining of lysosomes, was used to label autophagic compartments (Figure 8A). Incubation in the presence of AICAR increased (*p < 0.05*), whereas LH decreased (*p < 0.05*), amount of autophagosomes in luteal cells as evidenced by Cyto-ID staining (Figures 8B,C). Little co-localization with luteal lipid droplets was observed.

**FIGURE 9:**
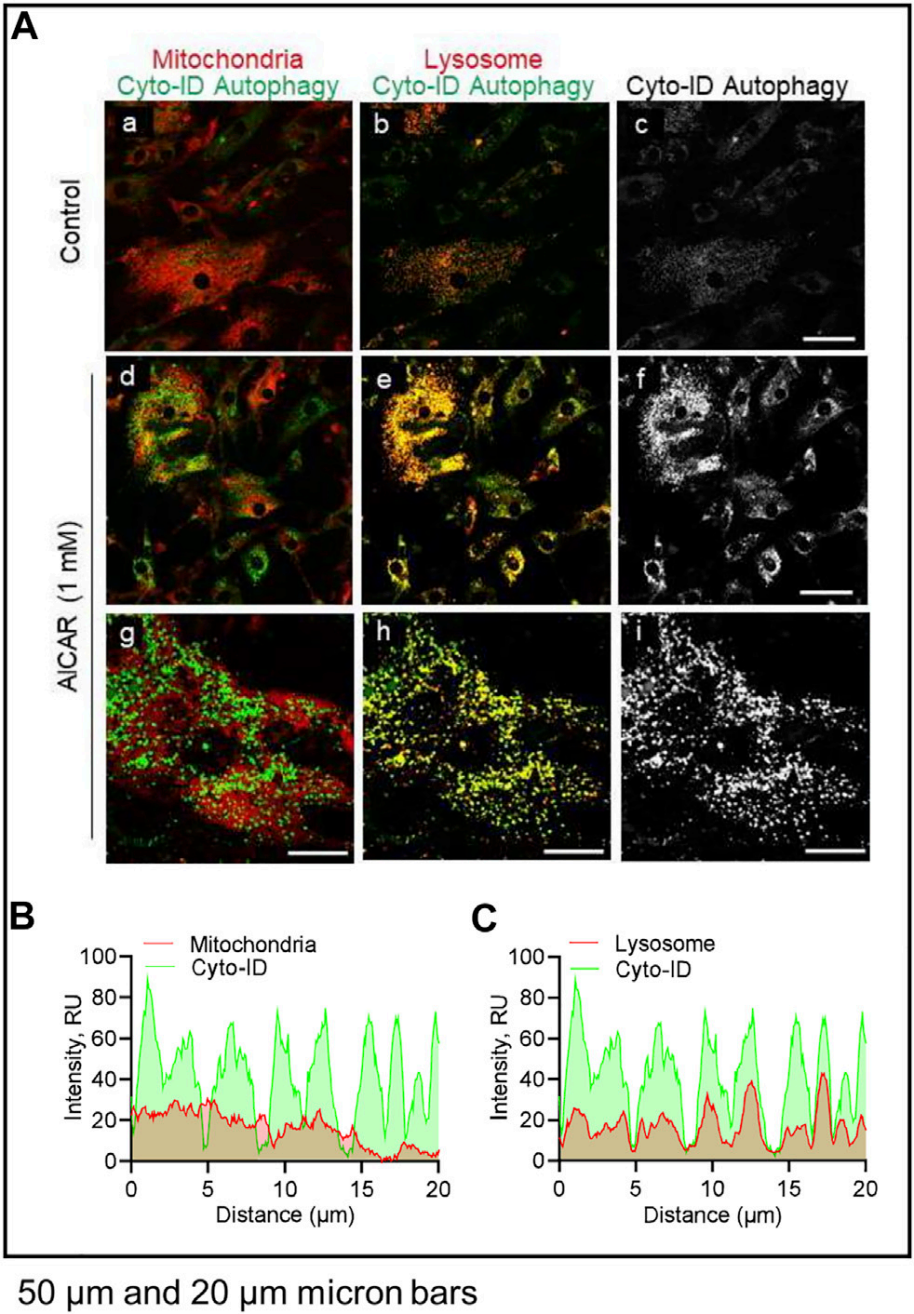
Effects of AICAR on localization of intracellular autophagic vacuoles *in vitro*. Luteal cells were stimulated with AICAR (1 mM) for 3 h and subject to confocal microscopy. **(A)** Representative micrographs showing the effects of AICAR on autophagy in luteal cells. From left to right; Merge of Mitochondria with Cyto-ID autophagy (Mitotracker; panels a, d and g), Merge of Lysosomes with Cyto-ID autophagy (Lysotracker; panels b, e and h) and Cyto-ID (white; panels c, f and i) obtained from cells treated (from top to bottom) with vehicle control or AICAR (1 mM). White box represents localization of intensity profile. **(B)** Intensity profiles illustrating the colocalized overlap of mitochondria (red) and Cyto-ID reagent (green). **(C)** Intensity profiles illustrating the colocalized overlap of lysosomes (red) and Cyto-ID reagent (green). Micron bars represents 50 and 20 µm.

LH did not affect co-localization of lysosomes or mitochondria with autophagosomes (not shown). In contrast, in cells treated with AICAR there was strong co-localization of lysosomes (lysotracker) and autophagosomes (Cyto-ID), but not mitochondria (Mitotracker) and autophagosomes (Cyto-ID), reflecting enhanced autophagy in cells incubated in the presence of the AMPK activator (Figure 9).

### Activation of AMPK Abolished Luteinizing Hormone-Mediated Phosphorylation of AMP-Activated Protein Kinaseα, Regulatory Associated Protein of Mechanistic Target of Rapamycin Kinase, Unc-51-Like Kinase 1 and Beclin 1

**FIGURE 10:**
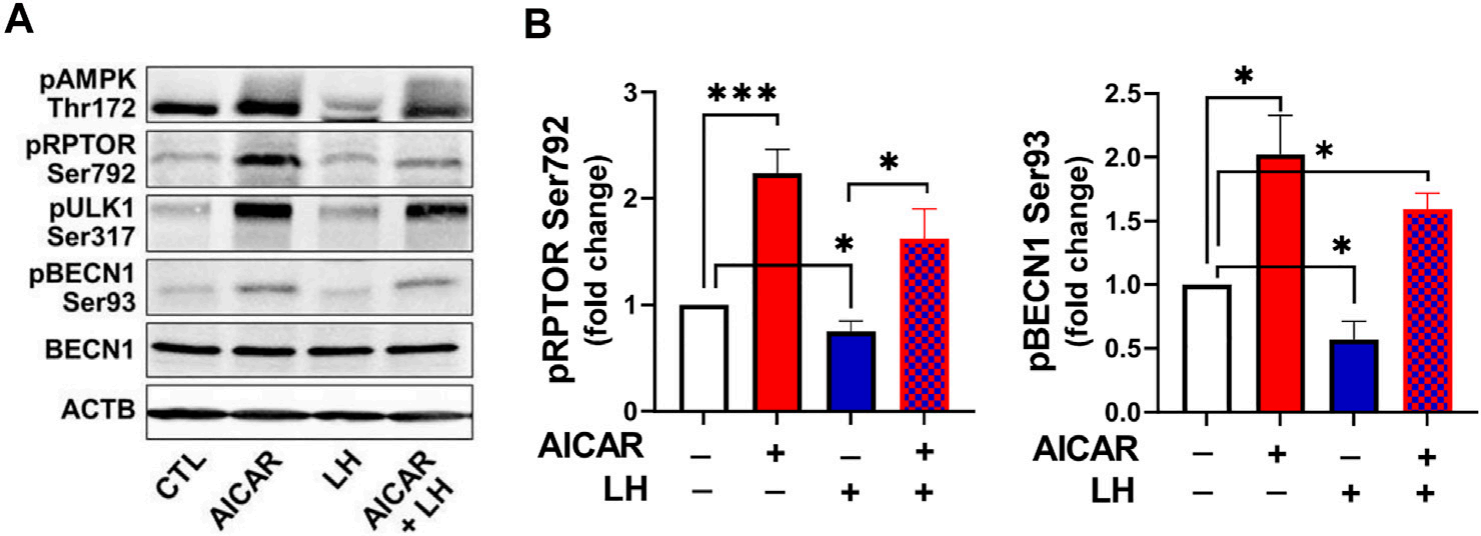
Activation of AMPK abolished LH-mediated phosphorylation of AMPKα, RPTOR and BECN1. Small luteal cells were pretreated with AICAR (1 mM) for 1 h and then treated with LH (10 ng/ml) for 4 h. Representative blots **(A)** and bar graphs **(B)** showing phosphorylation of AMPKα (Thr172) and the AMPK substrate RPTOR (Ser792) and BECN1 (Ser93). Bar graphs show average fold changes ±SEM from 3 to 5 separate experiments. Data were normalized to β-actin. Statistical analysis was performed using one-way ANOVA test for repeated measurements and Bonferroni post hoc test. Significant differences are indicated with asterisks *, *** representing *p* < 0.05, *p* < 0.001.

Considering the opposite effects of AICAR and LH on proteins involved in autophagy initiation, we determined whether AMPK activation would alter the inhibitory effect of LH on activity of RPTOR and BECN1 (Figure 10). Primary bovine luteal cells were pretreated with AICAR for 1 h and then treated with LH for 4 h. Treatment with AICAR elevated (*p < 0.05*) phosphorylation on AMPKα, RPTOR, ULK1 and BECN1. Additionally, pretreatment with AICAR abolished LH-mediated effects on AMPKα (Thr172), RPTOR (Ser792), ULK1 (Ser317), and BECN1 (Ser93) (Figures 10A,B) demonstrating that LH and AMPK mediate opposite effects on proteins involved in autophagy initiation.

## Discussion

The present study provides new insight into the signaling pathways regulating autophagy in the highly steroidogenic cells of the corpus luteum. *In vivo* studies revealed evidence for induction of autophagy in the corpus luteum after injection of a luteolytic concentration of PGF2α, e.g., the rapid phosphorylation of AMPKα and elevation of the autophagosome marker LC3B. *In vitro* studies supported previous reports that PGF2α triggers AMPK via the CAMKK2 pathway in luteal cells (Bowdridge et al., 2015). The present study demonstrated stimulatory effects of AMPK on autophagosome induction (LC3B-II and Cyto-ID staining) and posttranslational modifications of proteins required for MTOR inactivation (RPTOR) and autophagy initiation ULK1 and BECN1. Conversely, LH via PKA/MTOR signaling mediated inhibitory effects on autophagy initiation and reduced LC3B-II and Cyto-ID staining, indicators of diminished autophagosome formation in luteal cells. This study reveals that LH/PKA/MTOR signaling and PGF2α/Ca^2+^/AMPK signaling exert opposing actions on autophagy and phosphorylation of proteins crucial for autophagy initiation in luteal cells.

AMPK is a heterotrimeric protein that shifts cellular metabolism to catabolism when ATP levels are low (high AMP:ATP ratio). AMP binds to the catalytic subunit and prevents deactivation by phosphatases and may also recruit upstream kinases that activate AMPK. AMPK is activated by kinases such as serine/threonine kinase 11 (commonly called LKB1) and CAMKK2, which phosphorylate AMPK at Thr172, and it is conversely deactivated by PKA-mediated phosphorylation at Ser485, as well as phosphatases (Herzig and Shaw 2018). Here, we reported that luteolytic PGF2α increased AMPKα phosphorylation at Thr172 in the corpus luteum at the time of regression. In cultured luteal cells, PGF2α-induced AMPK activation was ameliorated with CAMKK2 inhibitor, STO-609, which is in agreement with a previous report in bovine luteal cells (Bowdridge et al., 2015). One of the hallmarks of autophagy is cleavage of LC3 into LC3-I and LC3-II (Tanida et al., 2008). In the present study, we demonstrated increased content of luteal LC3B protein in response to PGF2α treatment *in vivo*. Previous studies reported that LC3B protein increased in late-stage bovine corpora lutea (Aboelenain et al., 2015), suggesting that autophagy occurs during induced as well as physiological luteal regression. Similar results have been demonstrated in the corpus luteum of rat and goat (Choi et al., 2011; Grzesiak et al., 2018; Wen et al., 2020). Although autophagy is considered a pro-survival process; we demonstrated that autophagy-related signaling is upregulated during luteolysis, a process that is accompanied by cellular death (Song et al., 2018; Denton and Kumar 2019). Choi et al. (Choi et al., 2011) demonstrated that after 24 h of PGF2α treatment, both LC3B and caspase 3 are increased in cultured rat luteal cells. Dysregulation of autophagy can result in cellular death, so it is possible that these processes are connected in the context of the regressing corpus luteum; however, more mechanistic studies are needed.

The protein kinases, AMPK and MTOR, regulate activity of autophagy related proteins in opposite ways (Yang and Klionsky 2009). AMPK can affect autophagy via inhibitory effects on mTORC1 activity by phosphorylation of RPTOR, one of the proteins in the MTOR1 complex. Phosphorylation of RPTOR at Ser792 generates a docking site for inhibitory 14-3-3 proteins and is required for AMPK-mediated inhibition of MTOR1 (Gwinn et al., 2008). In the present study, treatment with AICAR rapidly phosphorylated AMPKα (Thr172), at site required for AMPK activity, and increased phosphorylation of RPTOR at Ser792, a site crucial for MTOR1 inactivation. Pretreatment with compound C, an inhibitor of AMPK, or transfection with dominant negative AMPKα1 abolished stimulatory effects of AICAR on posttranslational modifications of RPTOR confirming that the actions of AICAR in luteal cells were specific for AMPK. Therefore, it seems likely that inhibition of MTOR activity is one step by which activation of AMPK in luteal cells leads to induction of autophagy. The importance of MTOR in the regulation of autophagy in the bovine corpus luteum was suggested previously by Aboelenain and coworkers, who determined decreased MTOR expression and elevated levels of genes related to autophagy in the bovine corpus luteum during regression (Aboelenain et al., 2015).

Autophagy is a dynamically progressing process, which includes initiation, expansion, formation of autophagosomes, and fusion of autophagosomes to the lysosome. The ULK1/ATG13/FIP200 complex is a prominent initiator of autophagy in mammalian cells (Chan et al., 2009). As a serine/threonine kinase, ULK1 regulates autophagy by phosphorylation of its substrates such as BECN1, vacuolar protein sorting 34 (VPS34), autophagy related protein 13 (ATG13) and ATG101. ULK1 can be phosphorylated by AMPK at Ser317 or Ser555 to promote autophagosome formation (Kim et al., 2011). AMPK also contributes to autophagy induction by the phosphorylation of BECN1 at Ser90 or Ser93, a protein belonging to the class 3 phosphatidylinositol 3-kinase (PI3K) complex which is crucial for autophagy initiation (Chan et al., 2009). In the present study, activation of AMPK with AICAR increased phosphorylation of ULK1 Ser317 and BECN1 Ser93, whereas specific inhibitors of AMPK abolished the stimulatory effects of AICAR on phosphorylation of both proteins. Treatment with AICAR also increased content of LC3B-II, increased staining for autophagosomes (Cyto-ID), and stimulated colocalization of autophagosomes with lysosomes in luteal cells. Thus, we believe that AMPK can induce autophagy in the luteal cells by multiple routes including posttranslational modification of proteins belonging to two independent protein complexes fundamental for autophagy initiation.

The MTOR1 complex (MTORC1) comprises MTOR, mLST8 (or GβL), PRAS40, and RPTOR. Previous studies demonstrate that gonadotropins can activate MTOR (Alam et al., 2004; Hou et al., 2010). In this report, we confirm previous reports that LH and PKA activators stimulate MTOR and two known MTOR1 substrates. We observed that treatment with LH enhances phosphorylation of MTOR at Ser2448 and stimulation of the phosphorylation of p70S6K and ULK1. Inhibition of PKA abolished stimulatory effects of LH on p70S6K and ULK1, confirming that LH/PKA signaling can evoke MTOR1 activity in luteal cells (Hou et al., 2010). In the present study, LH stimulatory effects on phosphorylation of ULK1 at Ser757 were abrogated by pretreatment with rapamycin, an inhibitor of MTOR1, confirming that the effects on p70S6K and ULK1 were specific for MTOR1. Phosphorylation of ULK1 at Ser757 disrupts interaction between ULK1 and AMPK preventing autophagy initiation (Kim et al., 2011). Furthermore, we observed that LH/PKA signaling inhibited AMPK and RPTOR, which could contribute to enhanced MTORC1 activity and reduced autophagy. In fact, we observed that LH decreased basal and chloroquine stimulated content of LC3B-II as well as CYTO-ID staining of autophagosomes in luteal cells. Furthermore, treatment with dcAMP reduced number of autophagic vacuoles and phosphorylation of LC3B, required for autophagosomes formation in neuronal cells (Cherra et al., 2010). Thus, multiple molecules are activated in luteal cells in response to LH/PKA/MTOR signaling that individually or collectively contribute to the inhibition of autophagy. Further experiments are required to support the relative roles of molecules in luteal formation, maintenance and regression.

**FIGURE 11:**
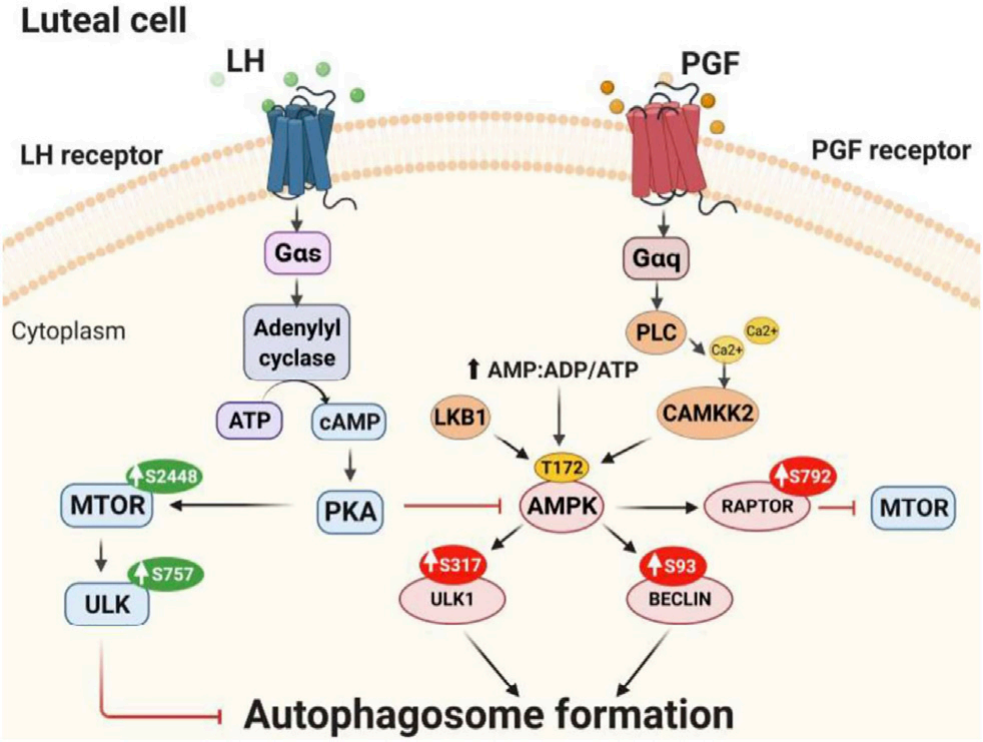
Proposed model of LH/PKA/MTOR and PGF2α/AMPK signaling in luteal cells. LH binds to G-protein coupled receptors present on the surface of luteal cells leading to increased activity of protein kinase A (PKA). LH/PKA increases the phosphorylation at Ser2448 (S2448) and activity of MTOR. Active MTOR phosphorylates ULK1 at Ser757 leading to its inactivation and preventing autophagosome initiation. Simultaneously, LH reduces phosphorylation of AMPKα at Thr172 (T172) and RPTOR at Ser792 (S792) maintaining MTOR activity and preventing autophagy induction. AMPK can be triggered by PGF2α or other stimuli that mobilize calcium or disrupt luteal metabolism. AMPK is activated by phosphorylation at Thr172 by upstream kinases [Calcium/Calmodulin-Dependent Protein Kinase Kinase 2 (CAMKKβ), Liver Kinase B1 (LKB1)] or by increased ratio of AMP/ATP. Activated AMPK phosphorylates RPTOR at Ser792 and inhibits MTOR activity. Active AMPK also phosphorylates ULK1 at Ser317 and BECN1 at Ser93 leading to autophagosome formation and autophagy in the corpus luteum.

In summary, the findings support the conclusion that LH/PKA/MTOR signaling and PGF2α/Ca^2+^/AMPK signaling mediate opposite and dynamic posttranslational modifications of proteins crucial for autophagy initiation in bovine luteal cells (Figure 11). Activation of AMPK during luteolysis rapidly induces posttranslational changes in RPTOR, ULK1 and BECN1 that inactivate MTOR and initiate autophagy. Conversely, LH via cAMP/PKA signaling reduces activating posttranslational modifications of AMPK, while stimulating the activity of MTOR. At the same time, LH/cAMP/PKA signaling phosphorylates ULK1 at a site required for inactivation, which results in decreased content of autophagosomes in bovine luteal cells. These findings extend current knowledge on the role of PKA and AMPK signaling in highly steroidogenic luteal cells.

## Data Availability

The original contributions presented in the study are included in the article/Supplementary Material, further inquiries can be directed to the corresponding author.
