# Supplementary material for: Protein Kinase A and 5′ AMP-Activated Protein Kinase Signaling Pathways Exert Opposite Effects on Induction of Autophagy in Luteal Cells
Source: Front Cell Dev Biol. 2021 Nov 8;9:723563. doi: 10.3389/fcell.2021.723563 (PMC8607825; doi:10.3389/fcell.2021.723563)
Supplement: Supplementary file 1 [file Image1.PDF]

Supplementary Material

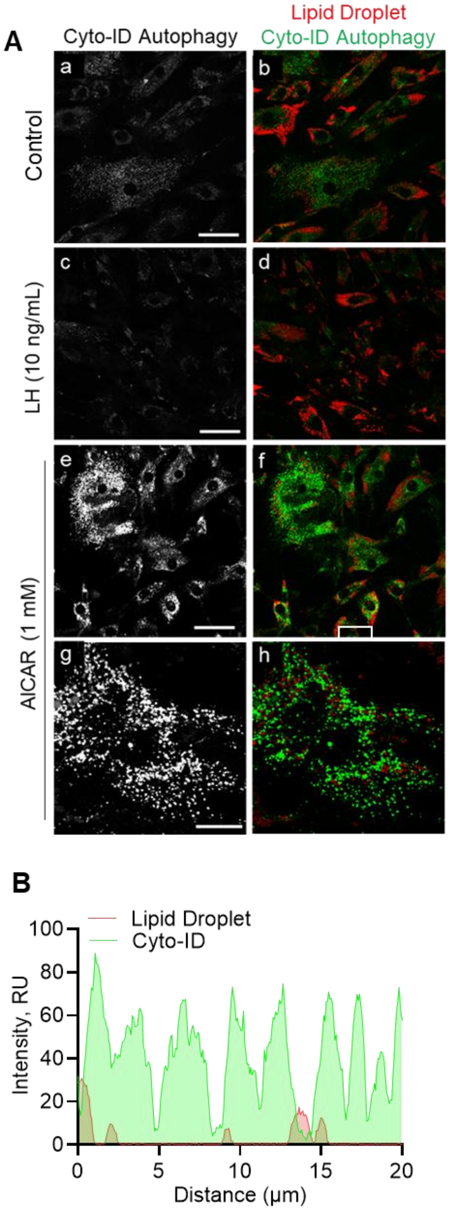

Supplemental Figure 1. Effects of (AICAR) on localization of intracellular autophagic vacuoles *in vitro*. Luteal cells were stimulated with AICAR (1 mM) for 3 h and subject to confocal microscopy. **(A)** Representative micrographs showing the effects of LH or AICAR on colocalization of Cyto-ID with lipid droplets in luteal cells. From left to right; Cyto-ID autophagy (white; panel a, c and e) and merge of Cyto-ID with Lipid droplets (Lipi-Blue; green; panel b, d and f) obtained from cells treated (from top to bottom) with vehicle control, LH (10 ng/mL) or AICAR (1 mM). White box represents localization of intensity profile. **(B)** Intensity profiles illustrating the colocalized overlap of lipid droplets (red) and Cyto-ID reagent (green). Micron bars represents 50 and 20 μm.
